# Minimally Invasive Surgical Techniques in Adult Degenerative Spinal Deformity: A Systematic Review

**DOI:** 10.1007/s11999-013-3441-5

**Published:** 2014-02-01

**Authors:** Konrad Bach, Amir Ahmadian, Armen Deukmedjian, Juan S. Uribe

**Affiliations:** Department of Neurosurgery and Brain Repair, University of South Florida, 2 Tampa General Circle, 7th Floor, Tampa, FL 33606 USA

## Abstract

**Background:**

Minimally invasive surgery (MIS) approaches have the potential to reduce procedure-related morbidity when compared with traditional approaches. However, the magnitude of radiographic correction and degree of clinical improvement with MIS techniques for adult spinal deformity remain undefined.

**Question/purposes:**

In this systematic review, we sought to determine whether MIS approaches to adult spinal deformity correction (1) improve pain and function; (2) reliably correct deformity and result in fusion; and (3) are safe with respect to surgical and medical complications.

**Methods:**

A systematic review of PubMed and Medline databases was performed for published articles from 1950 to August 2013. A total of 1053 papers were identified. Thirteen papers were selected based on prespecified criteria, including a total of 262 patients. Studies with limited short-term followup (mean, 12.1 months; range, 1.5–39 months) were included to capture early complications. All of the papers included in the review constituted Level IV evidence. Patient age ranged from 20 to 86 years with a mean of 65.8 years. Inclusion and exclusion criteria were variable, but all required at minimum a diagnosis of adult degenerative scoliosis.

**Results:**

Four studies demonstrated improvement in leg/back visual analog scale, three demonstrated improvement in the Oswestry Disability Index, one demonstrated improvement in treatment intensity scale, and one improvement in SF-36. Reported fusion rates ranged from 71.4% to 100% 1 year postoperatively, but only two of 13 papers relied consistently on CT scan to assess fusion, and, interestingly, only four of 10 studies reporting radiographic results on deformity correction found the procedures effective in correcting deformity. There were 115 complications reported among the 258 patients (46%), including 37 neurological complications (14%).

**Conclusions:**

The literature on these techniques is scanty; only two of the 13 studies that met inclusion criteria were considered high quality; CT scans were not generally used to evaluate fusion, deformity correction was inconsistent, and complication rates were high. Future directions for analysis must include comparative trials, longer-term followup, and consistent use of CT scans to assess for fusion to determine the role of MIS techniques for adult spinal deformity.

## Introduction

Minimally invasive (MIS) spine surgery has recently been at the forefront of innovations in spine surgery [4, 6, 9, 10, 13, 15, 16, 30, 31]. MIS spine surgery not only implies one performed through a smaller incision, but also an approach that seeks to reduce approach-related morbidity associated with traditional open spine surgery.

Traditional open surgical correction of adult degenerative scoliosis can be associated with perioperative risk and a prolonged recovery period [8, 12, 17, 19, 20, 29, 40]. Perioperative morbidity is compounded by the complexity of patients with adult degenerative scoliosis as well as patient-specific comorbidities [11, 21, 26, 27, 32]. MIS techniques, in comparison to open traditional surgery, may reduce approach-related and overall morbidity and so are attractive both to patients and surgeons [1, 18, 31, 33, 38]. However, the magnitude of radiographic correction and degree of clinical improvement with MIS techniques for adult spinal deformity remain ill defined. Moreover, minimally invasive techniques have a learning curve and pose their own set of unique challenges, technical limitations, and complications [2, 13, 14, 41].

We therefore sought to systematically review the available literature on MIS approaches for adult spinal deformity, specifically to determine whether they (1) improve pain and function; (2) reliably correct deformity and result in fusion; and (3) are safe with respect to surgical and medical complications.

## Materials and Methods

### Literature Review

A systematic PubMed and Medline database search was performed for published articles related to MIS techniques addressing adult degenerative scoliosis. MIS herein denotes an alternative surgical technique with the specific aim of reduced iatrogenic tissue damage incurred during the exposure process as compared with conventional open surgical approaches, thereby seeking to reduce perioperative morbidity. The search was limited to clinical studies in the English language from 1950 to August 2013 with the following key terms: “minimally invasive”, “surgery”, “adult”, “spine”, “deformity”, and “scoliosis”. The search criteria are detailed subsequently:

- Minimally [All Fields] AND invasive [All Fields] AND (“scoliosis” [MeSH Terms] OR “scoliosis” [All Fields])
- Minimally [All Fields] AND invasive [All Fields] AND “deformity” [All Fields])
- Minimally [All Fields] AND invasive [All Fields] AND (“scoliosis” [MeSH Terms] OR “scoliosis” [All Fields]) AND (“adult” [MeSH Terms] OR “adult” [All Fields])
- Minimally [All Fields] AND invasive [All Fields] AND “deformity” [All Fields]) AND (“adult” [MeSH Terms] OR “adult” [All Fields])

**Fig. 1:**
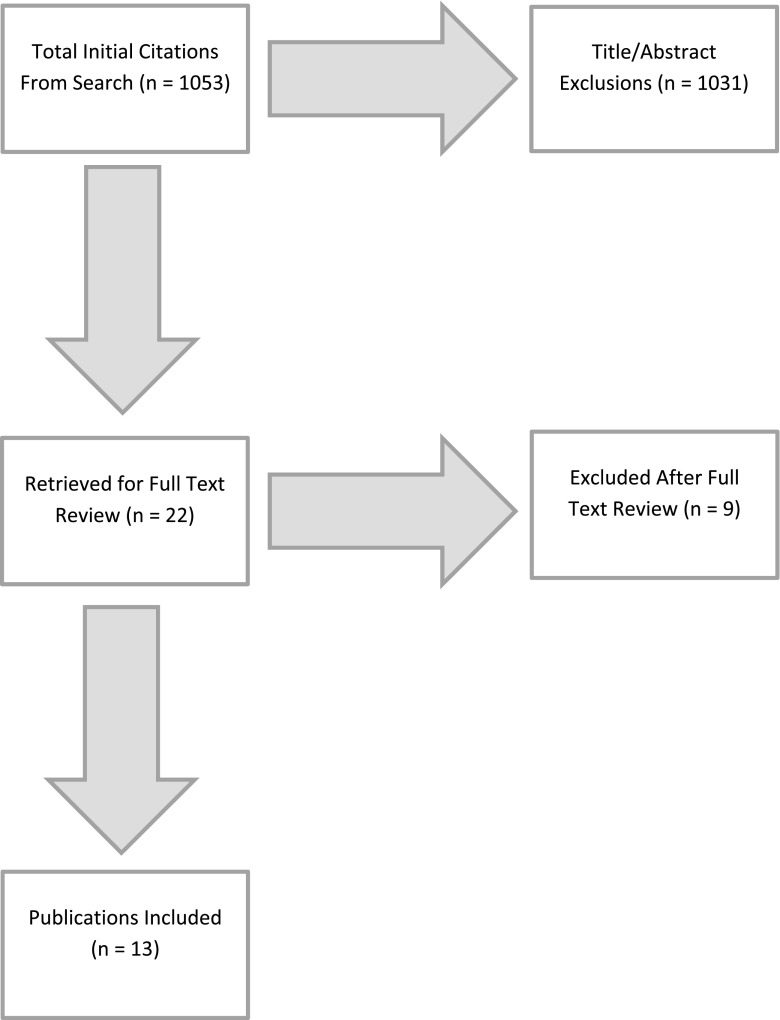
The figure illustrates the sequence of the literature search.

The searches yielded 147, 527, 77, and 302 papers, respectively (Fig. 1). Titles and abstracts generated by the search were subsequently reviewed and manuscripts were excluded from full-text review according to the following exclusion criteria: anatomical descriptions, case reports, commentaries, literature reviews, and studies addressing congenital or adolescent idiopathic scoliosis. Given the extremely limited data on MIS treatment of adult degenerative scoliosis, minimum followup was not one of the inclusion/exclusion criteria. We included papers with short-term followup to capture early complications.

Subsequently, the quality of the selected studies was judged by using the methodological index for nonrandomized studies (MINORS). The studies were further evaluated by the coauthors using the following criteria: completeness of operative data and perioperative complications, use of 36-inch standing radiographs, application of the current understanding of sagittal balance and spinopelvic parameters, length of followup ≥ 12 months (again, we used this as a criterion for evaluating study quality; studies with shorter followup were included to be able to identify early complications), reporting of a minimum of two health-related quality-of-life (HRQoL) questionnaires (eg, visual analog scale [VAS] and Oswestry Disability Index [ODI]), and statistical analysis for significance of radiographic and HRQoL outcome data.

Included studies were reviewed by each of the four authors. Disagreements were adjudicated by consensus.

The initial search yielded 1053 results. After title and abstract review, 22 papers were selected for full-text review. Of these, 13 were selected for inclusion in the review with a total of 262 patients. All studies were Level IV evidence: 11 retrospective case series, one prospective case series, and one retrospective cohort. MINORS [36] scores ranged from 5 to 10 for the case series and 13 for the study that included a control (Table 1). There were no prospective controlled or randomized trials.

**Table 1:** Summary of MINORS scores for papers included in the review

Study	MINORS score
Anand et al., 2008 [5]	8
Benglis et al., 2008 [9]	6
Anand et al., 2010 [6]	9
Dakwar et al., 2010 [13]	8
Tormenti et al., 2010 [39]	8/13*
Wang and Mummaneni, 2010 [43]	9
Isaacs et al., 2010 [25]	10
Marchi et al., 2012 [30]	8
Wang, 2012 [42]	5
Deukmedjian et al., 2012 [16]	8
Deukmedjian et al., 2013 [15]	10
Anand et al., 2013 [4]	9
Caputo et al., 2012 [10]	9

* The MINORS scoring system includes additional items for studies with control arms. This was the only study to include a control arm. For the sake of comparison with the other studies, the score for the first eight items was included followed by the total score for all items; MINORS = methodological index for nonrandomized studies.

### Patient Selection

Patient age ranged from 20 to 86 years with a mean of 65.8 years (Table 2). Inclusion and exclusion criteria were variable between studies. All required at minimum a diagnosis of adult degenerative scoliosis and some level of disability with one study also including a small number of patients with idiopathic scoliosis and iatrogenic scoliosis [5]. Only one study described specific indications for MIS rather than open treatments of their patients: those who had relative contraindications to a posterior approach, that is, previous surgery, age older than 65 years, and presence of comorbidities were treated with an MIS approach [26]. One group of authors attempted to delineate a schema for MIS modality selection based primarily on severity of sagittal plane deformity [15].

**Table 2:** Summary of patient age and diagnosis

Study	Mean age (years; range)	Diagnosis
Anand et al., 2008 [5]	72.8 (50–85)	Lumbar degenerative scoliosis
Benglis et al., 2008 [9]	58.8 (49–75)	Adult degenerative scoliosis
Anand et al., 2010 [6]	67.7 (22–81)	Degenerative lumbar scoliosis
Dakwar et al., 2010 [13]	62.5 (35–77)	Adult degenerative scoliosis
Tormenti et al., 2010 [39]	60 (48–69)	Adult degenerative scoliosis
Wang and Mummaneni, 2010 [43]	64.4 (42–84)	Adult degenerative scoliosis
Isaacs et al., 2010 [25]	68 (45–87)	Adult degenerative scoliosis
Marchi et al., 2012 [30]	71.8 (55–80)	Adult degenerative scoliosis
Wang, 2012 [42]	73 (62–80)	Adult degenerative scoliosis
Deukmedjian et al., 2012 [16]	64.7 (58–71)	Adult degenerative scoliosis
Deukmedjian et al., 2013 [15]	61 (32–74)	Adult degenerative scoliosis
Anand et al., 2013 [4]	64 (20–84)	Degenerative scoliosis (54), idiopathic scoliosis (11), iatrogenic scoliosis (6)
Caputo et al., 2012 [10]	65.9 (53–76)	Adult degenerative scoliosis
Average	65.8 (20–85)	

## Results

### Clinical Outcomes

Improvement in mean VAS leg and back pain scores ranged from 17.3 to 39.6 [4, 15, 30, 43] (Table 3). In the one study using this metric, the mean treatment intensity scale improvement was 18.9 (p = 0.009) [4]. Improvement in ODI scores was demonstrated in three studies, ranging from 9.3% to 33% [4, 15, 30]. Improvement in SF-36 scores was reported in one study, 20.9 (p = 0.01) [4, 5]. Minimum followup was 1.5 months (mean, 12.1 months; range, 1.5–39 months) in the 10 studies reporting quality-of-life outcomes [4–6, 10, 13, 15, 16, 30, 39, 43]. Quality-of-life outcomes were not reported in three studies [9, 10, 25].

**Table 3:** Summary of self-completed healthcare-related quality-of-life questionnaire outcomes

Study	Mean followup (months; range)	ΔVAS	ΔTIS	ΔODI	ΔSF-36
Anand et al., 2008 [5]	2.5 (0.5–4.7)	23	28	NR	NR
Benglis et al., 2008 [9]	10 (8–11)	NR	NR	NR	NR
Anand et al., 2010 [6]	22 (13–37)	40.2	27.62	32.1	5.8
Dakwar et al., 2010 [13]	11 (3–20)	57	NR	23.7	NR
Tormenti et al., 2010 [39]	10.5 (3–16)	33	NR	NR	NR
Wang and Mummaneni, 2010 [43]	13.4 (6–34)	27.8/39.6* (p < 0.01/p < 0.01)	NR	NR	NR
Isaacs et al., 2010 [25]	1.5	NR	NR	NR	NR
Marchi et al., 2012 [30]	6	51/35* (p < 0.001/p = 0.006)	NR	33 (p < 0.001)	NR
Wang, 2012 [42]	NR	NR	NR	NR	NR
Deukmedjian et al., 2012 [16]	9	26	NR	18	NR
Deukmedjian et al., 2013 [15]	17.4 (12–41)	28.7	NR	20	NR
Anand et al., 2013 [4]	39 (24–60)	17.3 (p < 0.001)	18.9 (p = 0.009)	9.3 (p = 0.006)	20.9 (p = 0.01)
Caputo et al., 2012 [10]	14.3	NR	NR	NR	NR

* Leg/back; VAS = visual analog scale; TIS = treatment intensity scale; ODI = Oswestry Disability Index; NR = not reported.

### Radiographic Results

Only four studies reported improvement in coronal Cobb angle from pre- to postoperative measurements [5, 10, 15, 39]; the range of improvement in these studies was 11° to 28.5° (Table 4). Only seven of the 13 studies used full-length standing radiographs to make this evaluation. Improvement in mean preoperative to mean postoperative coronal balance (cervicosacral vertical line) was demonstrated in one study, 14.5 cm (p < 0.0001) [4]. A mean improvement in apical vertebral translation of 12 cm (p < 0.001) and 14.1 cm (p < 0.001) was found in two studies [4, 10]. Sagittal vertical axis improved in two [4, 30] of the four studies [4, 15, 16, 30] that measured it (14.9 cm; p = 0.006 and 2.1 cm; p < 0.001 improvements, respectively) [4, 30]. Mean change in pelvic tilt was measured in three studies [15, 16, 30]. Only one study demonstrated an improvement of 11.4° (p = 0.009) [30]. Mean change in lumbar lordosis was measured in seven studies [10, 15, 16, 30, 39, 42, 43]. Three studies found an increase in lumbar lordosis of 7° (p = 0.02), 5° (p = 0.01), and 25.1° (p < 0.001) [10, 16, 30].

**Table 4:** Summary of radiographic outcomes

Study	36’ radiographs	ΔCC	ΔCSVL	ΔAVT	ΔSVA	ΔPT	ΔLL
Anand et al., 2008 [5]	No	12.74	NR	NR	NR	NR	NR
Benglis et al., 2008 [9]	No	10.2	NR	NR	NR	NR	NR
Anand et al., 2010 [6]	No	15	NR	NR	NR	NR	NR
Dakwar et al., 2010 [13]	No	14.85	NR	NR	NR	NR	NR
Tormenti et al., 2010 [39]	Yes	28.5 (p < 0.0001)	NR	18 (p = 0.031)	NR	NR	−6.9
Wang and Mummaneni, 2010 [43]	No	20	NR	NR	NR	NR	8
Isaacs et al., 2010 [25]	No	NR	NR	NR	NR	NR	NR
Marchi et al., 2012 [30]	Yes	NR	NR	NR	14.9 (p = 0.006)	11.4 (p = 0.009)	25.1 (p < 0.001)
Wang, 2012 [42]	Yes	27	NR	NR	NR	NR	21
Deukmedjian et al., 2012 [16]	Yes	NR	NR	NR	4.9	7	24
Deukmedjian et al., 2013 [15]	Yes	12/11/22* (p < 0.001/p = 0.001)	0.2/−0.7/1.5	NR	−0.6/1.4/0.1	−1/1/1	1/7/15 (p = 0.02)
Anand et al., 2013 [4]	Yes	15.2 (p < 0.001)	14.5 (p < 0.001)	12 (p < 0.001)	2.1 (p < 0.001)	NR	NR
Caputo et al., 2012 [10]	Yes	14.6 (p < 0.001)	NR	14.1 (p < 0.001)	NR	NR	5 (p = 0.01)

Angular measurements in degrees; linear measurements in centimeters; *patients divided into mild/moderate/severe deformity groups; CC = coronal cobb; CSVL = cervicosacral vertical line; AVT = apical vertebral translation; SVA = sagittal vertical axis; PT = pelvic tilt; LL = lumbar lordosis; NR = not reported.

Reported fusion rates ranged from 71.4% to 100% at 1 year postoperatively, but only two studies from 2013 used CT scans exclusively to assess arthrodesis [5, 16]. Use of bone graft and/or bone graft substitute to enhance fusion was reported in 10 of the 13 studies [4–6, 9, 10, 13, 15, 25, 39, 43]. Of these, seven used recombinant human bone morphogenetic protein 2 (rhBMP-2, Infuse; Medtronic, Memphis, TN, USA; Table 5 [4–6, 9, 13, 39, 43]).

**Table 5:** Biologics and fusion rates

Study	Biologics	Fusion rates/time/imaging
Anand et al., 2008 [5]	rhBMP2, Grafton Putty DBM	NR
Benglis et al., 2008 [9]	rhBMP2	100% 6 months, XR or CT
Anand et al., 2010 [6]	rhBMP2, Grafton Putty DBM	100 % 1 year, XR or CT
Dakwar et al., 2010 [13]	rhBMP2	80% 6 months, XR or CT
Tormenti et al., 2010 [39]	XLIF: AF versus DBM TLIF: IC versus BMP	NR
Wang and Mummaneni, 2010 [43]	BMP ± facet versus vertebral body versus iliac crest versus rib autograft versus allograft	100% interbody levels, 71.4% posterolateral levels without interbody fusion, fine-cut CT
Isaacs et al., 2010 [25]	Yes, not specified	NR
Marchi et al., 2012 [30]	NR	NR
Wang, 2012 [42]	NR	NR
Deukmedjian et al., 2012 [16]	Allograft	NR
Deukmedjian et al., 2013 [15]	NR	100% 1 year, CT
Anand et al., 2013 [4]	XLIF: rhBMP2, Grafton Putty DBM	94.4% 2 years, NR
	AxialLIF: rhBMP2, local autograft, DBM	
Caputo et al., 2012 [10]	Allograft cellular bone matrix	XLIF: 88.2% 1 year, CT; XLIF + ALIF: 90.9% 1 year, CT

AF = Actifuse bone graft; DMB = demineralized bone matrix; IC = autologous Iliac crest; BMP = bone morphogenic protein; XLIF = extreme lateral interbody fusion; TLIF = transforaminal lumbar interbody fusion; XR = dynamic radiograph; NR = not reported.

### Complications

Reported complication rates proved highly variable, ranging from 14.3% to 87.5% (Table 6). There were 115 complications reported among the 258 patients (46%), including 37 neurological complications (14%) (Table 7). One study did not report complications [9]. Aggregated complication rates (n = 258) were 3.9% (n = 10) motor, 10.5% (n = 27) sensory, 14.3% (n = 37) total neurologic, 6.2% (n = 16) infectious, 8.9% (n = 23) construct/hardware-related, 3.1% (n = 8) pulmonary, 3.5% (n = 9) cardiac, and 8.5% (n = 22) other perioperative complications. Of these, transient thigh paresthesias related to the lateral approach and wound infections were the most common.

**Table 6:** Summary of the minimally invasive surgical techniques in the review, operating room time, estimated blood loss, length of hospital stay, and complications encountered

Study	Number of patients	Technique	Operating room time (minutes)	Estimated blood loss (mL)	Hospital stay (days)	Complications (%)
Anand et al., 2008 [5]	12	XLIF or DLIF ± axial LIF L5-S1 + percutaneous pedicle screws	258/234*	171.9/92.5*	8.6	3 transient thigh dysesthesias, 1 transient thigh weakness (33)
Benglis et al., 2008 [9]	4	XLIF ± percutaneous pedicle screws	NR	NR	3.5	NR
Anand et al., 2010 [6]	28	XLIF or DLIF ± axial LIF L4-5 and/or L5-S1 + percutaneous pedicle screws	232/248*	241/231*	10	2 quadriceps palsies, 1 retrocapsular renal hematoma, 1 unrelated cerebellar hemorrhage (14.3)
Dakwar et al., 2010 [13]	25	XLIF ± lateral plate ± percutaneous pedicle screws	108/level	53/level	6.2	3 transient postoperative thigh numbness, 1 rhabdomyolysis requiring temporary hemodialysis, 1 asymptomatic subsidence, 1 asymptomatic hardware failure (24)
Tormenti et al., 2010^†^ [39]	8	XLIF ± TLIF + pedicle screws	NR	NR	NR	1 bowel perforation, 1 infection/meningitis, 6 postoperative sensory radiculopathy, 2 postoperative motor radiculopathy, 2 pleural effusion necessitating chest tube placement, 1 intraoperative hemodynamic instability, 1 pulmonary embolism, 1 ileus, 1 durotomy (during posterior stage) (50)
Wang and Mummaneni, 2010^‡^[43]	23	XLIF ± MIS-TLIF versus TLIF versus posterolateral fusion L4-5 L5-S1 + percutaneous pedicle screws	401	477	6.2	2 transient thigh numbness, 5 transient thigh numbness and pain, 1 persistent thigh pain and dysesthesias ipsilateral to approach, 1 sacroiliac joint pain syndrome, 1 pseudarthrosis at L1-2, 1 T-11 compression fracture at 12 months postoperatively, 1 CSF leak, 1 S1 screw pullout postoperative Day 34, revised with open operation, 1 asymptomatic afib postoperative Day 3, 1 pneumothorax (65)
Isaacs et al., 2010 [25]	107	XLIF ± axial LIF or posterior interbody approach for L5-S1 ± percutaneous versus open pedicle screws	178	50–100	2.8/8.1 (unstaged/staged)	1 MI, 1 sepsis 2° to UTI, 2 UTI, 2 afib, 2 hypotension requiring transfusion, 2 bacterial Infection, 1 postanesthesia delirium, 1 asymptomatic CHF, 1 pleural effusion, 1 hyponatremia, 1 pulmonary HTN, 1 GI bleed without transfusion, 3 posterior deep wound infection, 1 kidney laceration, 1 DVT, 7 motor deficit, 4 ileus, 2 pleural cavity violation requiring chest tube, 2 anemia requiring transfusion, 1 sensory deficit (33.6)
Marchi et al., 2012 [30]	8	MIS LIF with 20° or 30° lordotic cages ± percutaneous pedicle screws	210	131.3	NR	1 severe subsidence requiring revision at 3 months, 6 levels anterior endplate damage (87.5)
Wang, 2012 [42]	10	MIS TLIF + multilevel facet osteotomies + percutaneous pedicle screw + percutaneous iliac screws	302	480	5.6	1 patient with asymptomatic medial screw breach at T10 and L5, 1 epidural hematoma evacuated emergently with neurologic recovery (20)
Deukmedjian et al., 2012 [16]	7	MIS LIF with ALLR and 30° lordotic cage ± percutaneous pedicle and/or iliac screws	NR	125/530*	8.3 (5 days between stages of procedure)	1 superficial wound infection (14.3)
Deukmedjian et al., 2013 [15]	27 (divided into green, yellow, and red group)	MIS LIF ± ALLR ± axial LIF versus MIS TLIF L5-S1 ± percutaneous pedicle/iliac screws depending on group	NR	NR	NR	2 transient ipsilateral thigh numbness, 1 transient groin pain, 1 wound infection (14.8)
Anand et al., 2013 [4]	71	Stage 1: DLIF; Stage 2: axial LIF L4-5, L5-S1 ± percutaneous pedicle screws	Single stage: 412; two stage: 314/357	Single stage: 291; two stage: 183/243	7.6 (3 days between stages of procedure)	2 superficial wound infection, 4 pseudarthrosis, 3 radiculopathy, stenosis, 1 radiculopathy, heterotopic ossification, 1 delayed recurrent wound infection, 1 adjacent segment osteomyelitis, 1 adjacent segment discitis, 1 PJK, 1 proximal screw prominence, 1 idiopathic cerebellar hemorrhage (22.6)
Caputo et al., 2012^§^ [10]	30	XLIF + percutaneous pedicle screws L5-S1: ALIF	NR	NR	NR	1 lateral incisional hernia, 2 ALL rupture, 2 wound breakdown, 1 cardiac instability, 1 pedicle fracture, 1 nonunion requiring revision (26.6)

* Values given separately for AP component of surgery, respectively;^ †^this study also included a control group of 4 patients treated with either TLIF or PLIF for a total of 12 patients; ^‡^one patient had a revision procedure and was treated with open pedicle screws; ^§^only 30 of the 39 were included in outcomes analysis and only 22 of 39 were included in radiographic analysis; XLIF = extreme lateral interbody fusion; DLIF = direct lateral interbody fusion; LIF = lateral interbody fusion; ALLR = anterior longitudinal ligament release; NR = not reported; MIS = minimally invasive surgery; TLIF = transforaminal interbody fusion; CSF = cerebrospinal fluid; afib = atrial fibrillation; MI = myocardial infarction; UTI = urinary tract infection; CHF = congestive heart failure; HTN = hypertension; GI = gastrointestinal; DVT = deep vein thrombosis; PJK = proximal junctional kyphosis; ALL = anterior longitudinal ligament.

**Table 7:** Summary of complications data

Complication	Number	Percent (n = 258 patients)
Neurologic	37	14.3
Motor	10	3.9
Sensory	27	10.5
Infectious	16	6.2
Construct/hardware-related	23	8.9
Pulmonary	8	3.1
Cardiac	9	3.5
Other	22	8.5
Total	115	44.6

## Discussion

With an ever-increasing array of surgical options, minimally invasive spine surgery techniques are altering treatment paradigms for a variety of spine disorders, including the complex field of adult degenerative scoliosis. In making the decision to treat a patient with a minimally invasive rather than traditional open technique, the surgeon must be aware of both the advantages and limitations of the selected surgery. This systematic review found the literature on MIS approaches to adult spinal deformity to be both scant and rather preliminary. In general, followup was short, comparator groups absent, and basic methodological approaches such as the use of CT scans to assess fusion and full-length radiographs to assess deformity correction to have been inconsistently used. Although scores for pain and function generally improved, radiographic improvements were more modest and complications relatively frequent. Because, with the exception of one very small study, there were no comparisons made in these studies with traditional approaches, it is difficult to say whether these approaches delivered on the promise of safer or comparably effective surgery.

This study had a number of limitations. First, as noted, control groups were absent. This makes it impossible to determine whether the improvements observed are better than, worse than, or comparable to more traditional approaches. No comparisons could be made that control for levels of spine operated on or the number or combination of modalities used. Second, followup in these studies was very short, and this limits our ability to comment on either the durability of the corrections observed or the reoperation frequencies with any confidence. Only one study had greater than 2 years mean followup [4]. To establish MIS techniques as a useful tool in optimizing the surgical strategy and patient outcomes, a greater number of clinical studies demonstrating durable results is needed. Third, without CT scans, it is difficult to know for sure whether fusion has been achieved in these patients; only two of 13 studies used CT scans exclusively for the assessment of fusion. Fourth, this review is limited in its capacity to articulate the true incidence of complications. In analyzing complication rates, all complications were added together and then divided by the total number of patients. This type of analysis assumes a one-to-one correspondence of complications to patients not accounting for the possibility of multiple complications in a single patient, thus likely representing an overestimate of actual complication percentages; this may be offset by the fact that the followup was very short, and some complications (such as chronic and subacute complications such as pseudarthrosis or junctional disease [7, 44]) and reoperations will therefore not be well represented in the studies we surveyed. One particular study noted an alarmingly high complication rate of 87.5%. We believe that this rate stemmed from a technique-specific complication. The authors of this paper used hyperlordotic cages without release of the anterior longitudinal ligament. Thus, six of the eight patient cohort experienced anterior endplate damage, and one further patient developed asymptomatic cage subsidence.

**Fig. 2A–B:**
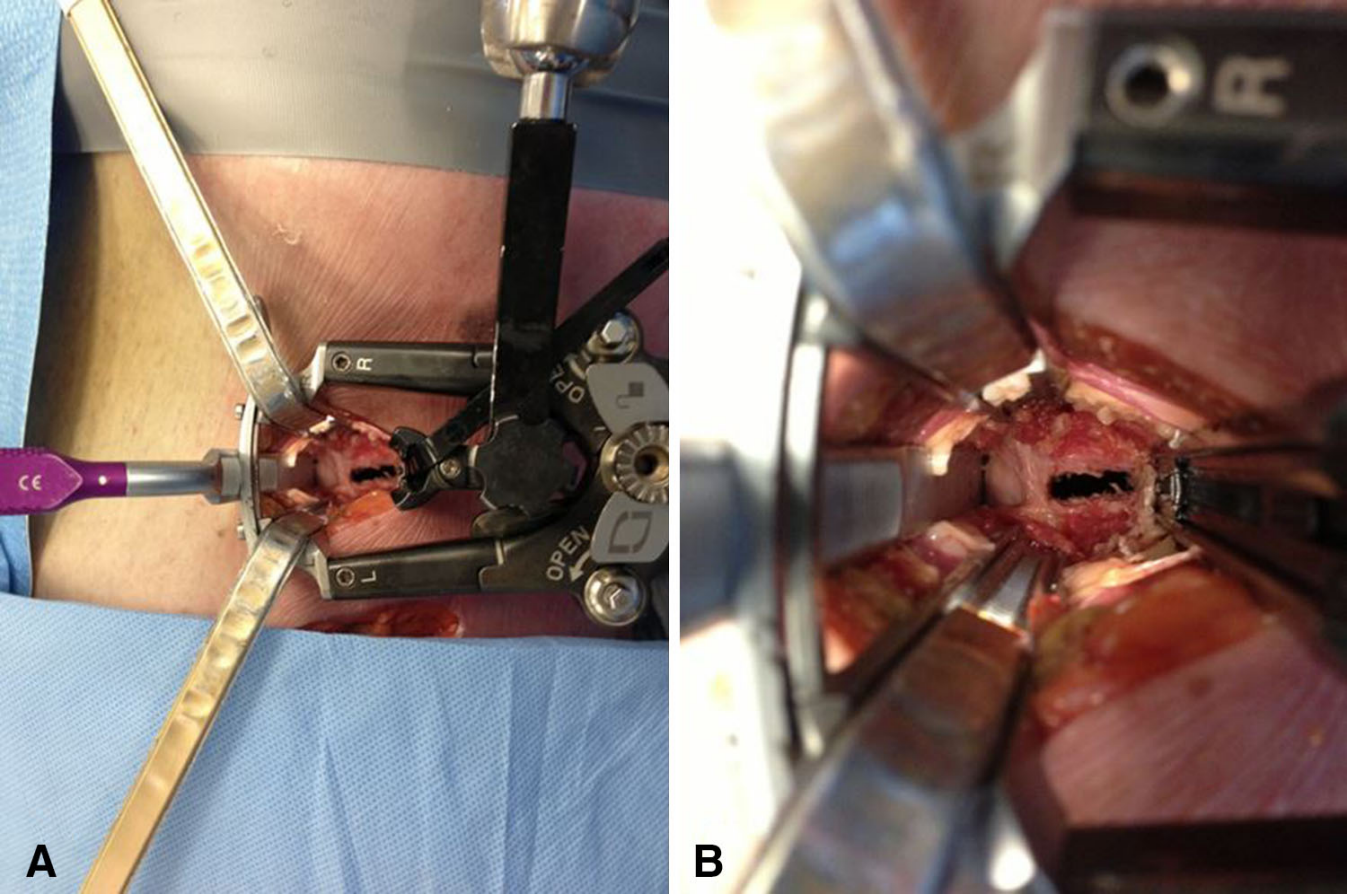
The figure demonstrates the live intraoperative view (**A**) and close-up (**B**) of the MIS lateral approach using XLIF^®^ (Nuvasive, San Diego, CA, USA).

**Fig. 3A–D:**
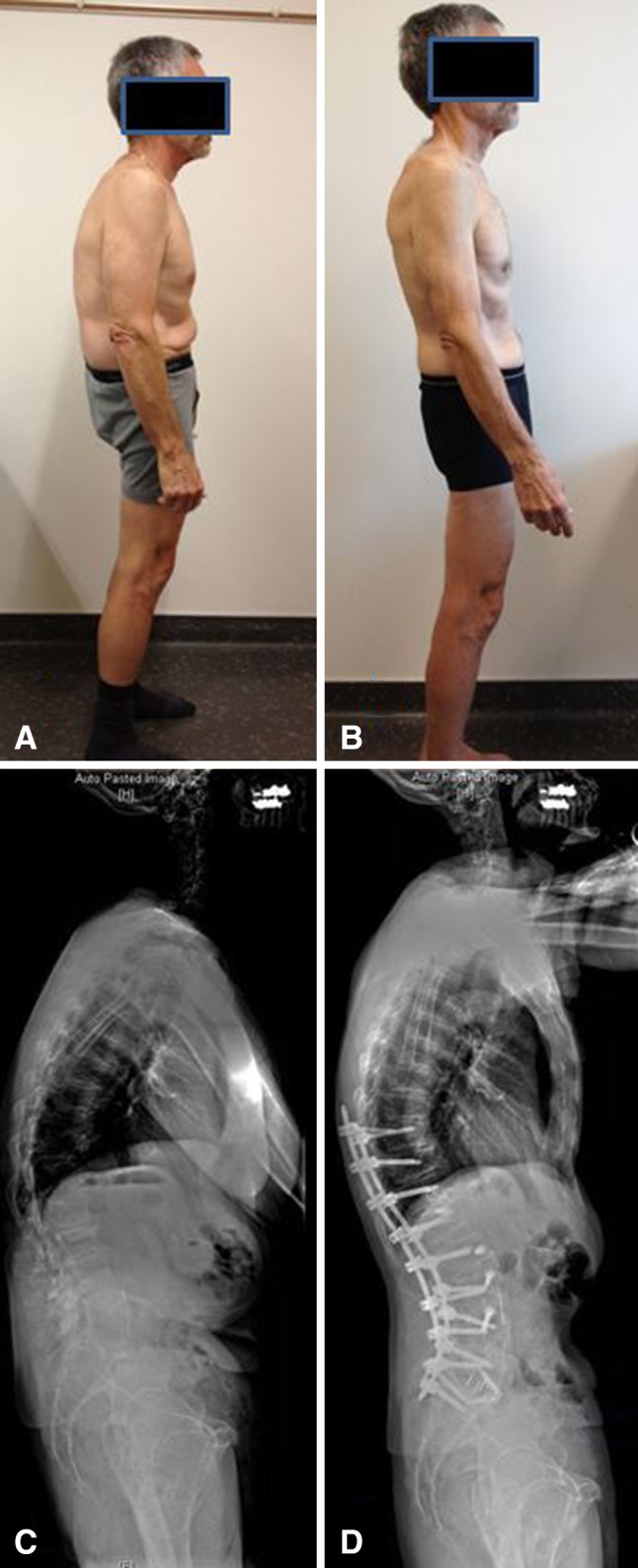
The patient is a 67-year-old man who initially presented to the clinic with severe back and leg pain and limited walking ability. Preoperative sagittal balance, ODI, and VAS back/leg pain were 116 mm, 50%, and 4.7, respectively. The patient was treated with the T10-L5 MIS lateral approach, L5-S1 ALIF, T12L1, L23, L34 anterior longitudinal ligament release with a hyperlordotic cage placed at those levels, and T10-S1 percutaneous pedicle screws. Two-year followup sagittal balance, ODI, and VAS were 34 mm, 2%, and 2, respectively. (**A**) Preoperative side-view photograph. (**B**) Postoperative side-view photograph. (**C**) Preoperative lateral radiograph. (**D**) Two-year followup lateral radiograph.

The specific MIS techniques described in the studies were generally separated into anterior column support and posterior instrumentation (Table 6). The approach to the anterior column, discectomy, and interbody fusion was accomplished using some variant of a minimally invasive far lateral approach. Examples include extreme lateral interbody fusion (Fig. 2) (XLIF^®^; Nuvasive, San Diego, CA, USA) and direct lateral interbody fusion (DLIF^®^; Medtronic). In addition to an MIS lateral interbody fusion, three studies added the use of hyperlordotic cages to increase correction of sagittal deformity and restore lumbar lordosis with or without the use of an anterior longitudinal ligament release (Fig. 3) [15, 16, 30]. As a result of anatomical constraints, the lumbosacral junction is not accessible through a far lateral approach necessitating the use of an alternate technique. Three MIS techniques were used either exclusively or alternately in multiple studies for interbody fusion at the lumbosacral junction and in certain cases at L4-5. These include AxialLIF^®^, MIS transforaminal lumbar interbody fusion (TLIF), and anterior lumbar interbody fusion. With the exception of one study that used lateral plates [13], instrumentation exclusively took the form of posterior percutaneous pedicle screw fixation. Beginning with Wang in 2012 [42], percutaneous screw fixation to the ilium demonstrated use in select cases. Of note, several studies included cases in which patients were treated with standalone interbody fusions without additional instrumentation [4, 9, 15, 25, 30].

### Clinical Outcomes

From the studies we reviewed, it appears the VAS scores for leg/back pain are emerging as the primary tools for assessing clinical outcomes. Unfortunately, from the variability in reporting, it was not possible to combine all patient data points into a single analysis. With the exception of Anand et al. [4], the studies in the review are limited both in terms of cohort size and mean length of followup. Higher powered studies with adequate control groups will be needed to fully elucidate the use of MIS techniques in improving patient outcomes.

### Radiographic Results: Deformity Correction and Fusion

Standing full-length radiographs are critical in the assessment of sagittal balance and spinopelvic parameters in a patient with adult degenerative scoliosis [3], but only half of the studies in the review obtained them. Of the studies that did, only Deukmedjian et al. [15] included a complete preoperative and postoperative measurement of all parameters of sagittal balance and spinopelvic harmony. Tormenti et al. was the only study to show a loss of mean lumbar lordosis; however, three patients in the cohort had hyperlordosis (> 60°) and one loss of lordosis [39]. Three studies included in their radiographic analysis reduction of apical vertebral translation [5, 10, 39]; however, to our knowledge, no evidence has been published in the literature demonstrating a correlation between apical vertebral translation and clinical outcome. Anand et al. did analyze sagittal balance but did not include any preoperative or postoperative analysis of spinopelvic parameters [5]. Restoration of sagittal balance and spinopelvic harmony has been demonstrated to be a strong predictor of functional outcome and represents a critical component in planning and evaluating deformity correction surgery [3, 23, 24, 28, 35, 37]. We hope that with more widespread awareness of the importance of spinopelvic parameters and availability of diagnostic technologies, there will be future studies analyzing global spinal balance. Furthermore, only two of the included 13 studies consistently used CT scans for assessment of fusion [10, 15]. CT scans provide exquisite bony detail compared with dynamic radiographs and have higher sensitivity for detecting early hardware failure. We hope that in future studies, use of CT scans for assessment of fusion will become more widespread.

Use of allograft has increased concomitantly with MIS techniques to enhance fusion rates. Ten of the 13 studies in the review used some form of biologic agent to enhance fusion rate [4–6, 9, 10, 13, 16, 25, 39, 43]. A recent systematic review and meta-analysis by Rodgers et al. using previously unavailable internal data from the manufacturer demonstrated enhanced fusion rates in patients undergoing anterior fusion supplemented with rhBMP-2 compared with iliac crest bone graft [34]. However, using the same data, Fu et al. found that fusion rates for rhBMP-2 were comparable [22]. Both studies did find an increased risk of genitourinary complications with the use of rhBMP-2 [22, 34]. At present, indications for the use of rhBMP-2 versus bone graft remain to be clarified [22, 34].

### Complications

Given both the limited cohort size and novelty of some of the techniques, some potential complications may not have been encountered. For example, advanced techniques such as anterior longitudinal ligament release carry an identifiable risk of great vessel injury [16], although none were reported. Safety of the neural structures in deformity surgery is of paramount importance. At first glance, a 14.3% neurologic complication rate appears high. However, the majority of these are transient and related to the lateral approach. A thorough working knowledge of lumbar plexus anatomy and use of continuous electromyographic neuromonitoring should serve to minimize the incidence of these approach-related complications. One of the aims of MIS treatment is a reduction in perioperative complications in a population predisposed to comorbidity. Only Isaacs et al. reported 28.3% of patients having at least one comorbidity before surgery [25].

### Conclusions

At present, there exists a glaring paucity of studies investigating MIS treatment of adult degenerative scoliosis that meets the standards used for evaluating traditional deformity surgery. Work remains to be done in producing more robust studies with longer followup to determine durability of correction, subsidence rates, and improvement of quality of life. To make concrete claims about the efficacy of MIS treatment of deformity, studies with control groups treated with traditional deformity surgery are necessary. Consistent use of CT scans for assessment of fusion is needed because this is the main purpose of these surgical procedures. In addition, further study is needed to delineate the role of advanced techniques such as anterior longitudinal ligament release and use of hyperlordotic cages. Finally, given that adult degenerative scoliosis affects predominantly elderly patients, more data with larger cohorts fitting this demographic are needed to assess if MIS techniques reduce the incidence of age-related complications in patients undergoing spine surgery.
